# Documentation of anthropometrics in people with cancer: a cross-site collaboration audit in four hospital settings in the UK

**DOI:** 10.1007/s00520-024-08931-3

**Published:** 2024-10-10

**Authors:** F. Tabacchi, R. Oberai, K. Parmar, L. Oxley, S. Coe, V. Iatridi, J. Tammam, E. Watson, H. Wanstall

**Affiliations:** 1 Institute of Nursing, Midwifery and Allied Health Research (OxINMAHR), Oxford, UK; 2 Department of Dietetics, Therapies, Churchill Hospital, Oxford University Hospitals NHS Foundation Trust, Oxford, UK; 3 Nutrition and Dietetics, Therapies, Maidstone Hospital, Hermitage Lane, Maidstone, ME16 9QQ UK; 4 Department of Nutrition and Dietetics, Therapies, Heartlands Hospital, University Hospitals Birmingham, Birmingham, UK; 5 Nutrition and Dietetics Department, Queen Elizabeth Hospital, Mindelsohn Way, Edgbaston, Birmingham, B15 2GW UK

**Keywords:** Malnutrition, Dietetic referrals, Oncology, Nutrition support, Nutritional screening

## Abstract

**Background:**

Malnutrition is a significant risk for patients during cancer treatment. Neglecting to monitor or provide timely dietetic support can result in lower tolerance to treatments and reduced quality of life. This audit aimed to assess the completeness and accuracy of the documentation of anthropometric measurements in medical records and dietetic referral practices across four day-treatment units (DTUs) in England.

**Methodology:**

Data were collected from electronic patient records of 100 patients in each DTU attending for systemic anti-cancer treatment (SACT) over a 2-week period. Data collected included patients’ demographics, anthropometric data, referrals to dietitians, and whether the patients referred had a MUST score ≥ 2, which was calculated by the authors.

**Results:**

Findings revealed that weights and heights were documented for 58–85% and 94–98% of patients attending DTUs, respectively. On average, 55% (range of 7–85%) of patients had their body mass index (BMI) documented on the day of SACT. The Malnutrition Universal Screening Tool (MUST) was rarely completed (≤ 3% in each centre). Dietetic referral practices varied across centres.

**Conclusions:**

Findings highlight the need to improve anthropometric documentation practices in cancer centres, in order to allow better monitoring of malnutrition risk and early nutritional support interventions when needed.

## Introduction

Malnutrition is a widespread challenge in people with cancer, affecting up to 80% of people living with advanced disease [1]. Malnutrition is defined as a state of nutrition in which a deficiency or excess of energy, protein, and other nutrients causes measurable adverse effects on tissue and function [2]. It is associated with lower tolerance to treatments, higher mortality, and reduced quality of life, and higher costs for healthcare systems [3, 4]. Therefore, it is important to identify malnutrition and provide timely dietary support in cancer patients as part of their routine care, in line with current oncology European guidelines [5–8]. Anthropometric measurements such as body weight and body mass index (BMI) are recommended to be regularly monitored in cancer patients as they can help identify early risk of malnutrition. Weight is also used to calculate medication dosages, including systematic anti-cancer therapy (SACT) doses and weight trend of the last 6 months which can be used as part of the assessment of the patient’s overall fitness for treatment. However, several studies [5, 6] have shown that these measurements are not consistently performed or documented in clinical practice, despite being a simple and non-invasive way to identify patients at nutritional risk [7, 8]. Additionally, guidelines recommend the regular use of a malnutrition screening tool in cancer centres [9]. Malnutrition Universal Screening Tool (MUST) is the most commonly used tool in the UK and has been validated in several populations including cancer; however, there is a lack of consensus on which tool to use in cancer patients, leaving this decision to the individual centres [8, 10]. Empirical evidence suggests that even validated tools are not consistently utilised in practice.

An audit of electronic patient records of four different NHS Day Treatment Units (DTU) in England was conducted, aiming at providing a snapshot of the current situation in terms of accuracy and completeness of anthropometric documentation and dietetic care provision for oncology patients. The underlying goal was to verify whether documentation was complete enough to identify weight loss and allow timely referrals for nutrition support. A secondary aim of the study was to compare referral practices among different cancer centres.

## Methods

Data collection was conducted simultaneously at four cancer centres based in England. Two centres were in the South-East England and two in the Midlands. The DTUs participating in this audit treat from 100 to 300 oncological patients daily, of which 60 to 70 receive SACT.

Approval and registration of the audit were sought and obtained in each centre’s online register before data collection started. The registration numbers for centres A–D were 7882, 02110, 19,070 and 18,754, respectively. The study was also approved by Oxford Brookes University Health & Social Care Research Ethics Sub-Committee (HSCRES)—FHLS Ethics Number: HLS/2022/ZD/01. Permission to share anonymized data among the four centres to allow statistical analysis was also obtained from each centre.

All centres used an alphabetical randomisation to select the patients to include. The inclusion criterion was patients receiving Systematic Anti-Cancer Therapy (SACT) on the day of DTU attendance. Exclusion criteria were patients attending DTU for treatment other than SACT, patients who discontinued treatment on the appointment day or were admitted to hospital after receiving SACT and patients receiving private healthcare services. Each centre collected data for 100 cancer patients during 2 weeks in February 2023; a total of 400 patients (approximately 17% of the population attending DTU). Data were collected in a bespoke Excel 365 spreadsheet. All four centres made use of electronic medical records and used MUST (https://www.bapen.org.uk/pdfs/must/must_full.pdf) as malnutrition screening tool at the time of data collection. Demographics were noted including sex, age, ethnicity, and cancer diagnosis. Electronic records of each patient were assessed for whether height, weight, BMI, and MUST score were documented during the SACT visit to the DTU. The accuracy of the documented BMI and MUST score was also assessed through manual recalculation. BMI was re-calculated—or calculated in cases where it was not documented—using the formula BMI = weight (kg) ÷ height^2^ (meters) by each specialist dietitian based on data from their centre. MUST score was re-calculated—or calculated in cases where they were not documented—by trained specialist oncology dietitians using the ‘MUST’ Explanatory Booklet (https://www.bapen.org.uk/pdfs/must/must_explan.pdf) and their own clinical judgment. Step 1 (BMI score) was calculated by using the formula above. Step 2 (weight loss score) was calculated by using the percentage of unplanned weight loss in the last 3–6 months. Anthropometric data to perform these calculations were taken from patients’ electronic medical records. In cases where information needed was not documented on the data collection date, records were checked from the week before and after. If no data was documented within this period, BMI or MUST score was classified as ‘indeterminable’. Step 3 (acute disease effect score) was calculated according to clinical background, current medical issues, symptoms, treatments, and oral intake, as documented in the patients’ electronic medical records. Consensus was reached upon discussion among specialist dietitians in case of uncertainties.

Referrals to dietitians were noted and assessed for appropriateness: based on MUST score, considering MUST score ≥ 2 appropriate for dietetic referral. The authors calculated MUST score for all patients in order to assess the appropriateness of the referrals. This decision was taken to ensure standardisation across sites; the limitations of this approach are noted in the discussion. Patients who were identified as at risk for malnutrition (MUST ≥ 2) but not referred to the dietetics service were also noted. The data were audited against NICE standards, which recommend that 100% of outpatients with clinical concerns should be screened at each appointment or weekly if they have multiple appointments in a week [5]. Guidelines also suggest that all outpatients should have their height measured at least on their first appointment in clinic [3]. Where more than one height measurement was recorded, the consistency of the different measurements was checked. Inconsistent heights were defined as heights with > 5 cm difference uploaded at different times in the patient’s notes. In order to facilitate discussion and interpretation of the results, each centre also recorded the oncology dietetic team composition, estimated dietetic hours dedicated to DTU patients, and dietetic referral methods and criteria for their site.

## Results

The characteristics of the sample are shown in Table 1. The population from different cancer centres show a majority of female, white patients in each centre, an average age of 62 years old, and an average BMI of 27.5 kg/m^2^. Centres B and D have a separate DTU for haematological malignancy which is why they only collected data for solid tumours. Full detail of the population of each centre can be found in Appendix Table 4.

**Table 1:** Overview of key population characteristics of all cancer centres

Patient characteristic	No. (%)
Gender Female Male	247 (61.7) 153 (38.2)
Ethnicity—self-described White Other Prefer not to say Asian Black	178 (59.3) 48 (16.0) 42 (14.0) 20 (6.6) 12 (3.6)
Cancer diagnosis Breast LGI* Gynaecological Haematological HPB* Lung Pelvic Others* Head and neck Melanoma UGI*	78 (19.5) 73 (18.2) 43 (10.7) 43 (10.7) 40 (10.0) 33 (8.2) 22 (5.5) 19 (4.7) 17 (4.2) 17 (4.2) 15 (3.7)
Age range (median) in years	20–88 (62.0)
Weight range (median) in kg	32–127 (76.5)
BMI range (average) in kg/m^2^	13–46.7 (27.5)

*LGI, lower digestive tract; *HPB*, hepato-pancreato-biliary; *UGI*, upper gastrointestinal tract. Others include osteosarcomas, neuroendocrine, myelodysplastic syndromes, kidney, and unknown primary

Table 2 shows that 58–85% of patients’ weights were documented on the day of the patient attending DTU for SACT. In regard to BMI, manual recalculation based on documented weights and heights revealed that all patients from centres B, C, and D who had a weight and height measurement also had an accurate BMI recorded. Height was measured and documented for the majority of patients (94–98%) in all centres; however, 12–20% of the heights were documented inconsistently. Less than 3% of MUST scores were documented in all centres. The accuracy of BMI and MUST documentation was assessed by manual recalculation of these scores as described in methods and is illustrated in Table 2.

**Table 2:** Anthropometric and screening data documentation in different centres and overall percentages

	Centre A	Centre B	Centre C	Centre D	Total
Weight documented on the day of treatment—No. (%)	58 (58)	85 (85)	63 (63)	65 (65)	271 (67.7)
BMI documented on the day of treatment—No. (%)	7 (7)	85 (85)	63 (63)	66 (66)	221 (55.2)
BMIs documented correctly—No. (%)	6/7 (85.7)	85/85 (100)	63/63 (100)	66/66 (100)	220/221 (99.5)
Height documented in medical records—No. (%)	94 (94)	95 (95)	98 (98)	95 (95)	382 (95.5)
Height consistent*	80/94 (85.1)	95/95 (100)	79/98 (80.6)	84/95 (88.4)	338/382 (88.4)
MUST scores documented on day of treatment—No. (%)	2 (2)	3 (3)	0 (0)	1 (1)	6 (1.5)
MUST documented correctly (%)	1/2 (50)	2/3 (66.6)	0/0 (0)	1/1 (100)	4/6 (66.6)

*Values represent the number of patients with consistently documented height measurements, out of the total number of patients who had more than one height measurement recorded in their medical records. Consistent heights were defined as different heights with < 5 cm difference uploaded at different times in the patient’s notes

Dietetic referrals and their appropriateness were assessed and reported in Table 3. Centres A and B have almost double the amount of dietetic referrals compared to centres C and D. A very high percentage of patients (from 80 to 100%) was referred appropriately (according to the criteria MUST scores ≥ 2) across all centres despite the lack of screening, with all of the referrals from centres C and D deemed appropriate according to the criteria above. However, in all centres apart from B, a high number of patients should have been referred to dietetic care but were missed (1.1–18.3%).

**Table 3:** Dietetic referral rates and distribution in cancer centres at the time of the audit

	Centre A	Centre B	Centre C	Centre D	Total
Patients in this sample that had been referred to dietitians at the time of audit—No. (%)	20 (20)	21 (21)	9 (9)	9 (9)	59 (14.7)
Patients referred to dietitians appropriately*—No. (%)	16/20 (80)	18/21 (85.7)	9/9 (100)	9/9 (100)	52/59 (88.1)
Patients who should have been referred to dietitians but were not—No. (%)	16/87 (18.3)	1/87 (1.1)	13/96 (13.5)	20/92 (14.1)	50/362 (13.8)
Patients for whom it was not possible to say whether they needed a dietitian due to lack of data—No. (%)	13 (13)	13 (13)	4 (4)	8 (8)	38/400 (9.5)
Ratio of referrals coverage**—%	55.6	95.5	40.9	31	0.57

*Patients referred to dietitians and having a MUST score of ≥ 2 deemed as appropriate

**No. of patients referred/(No. of patients referred appropriately + patients who should have been referred but were not)

The authors were not able to calculate MUST scores for 9.5% of the sample due to lack of documentation. Consequently, it was not possible to determine whether these cases represented missed referrals or not.

## Discussion

This study provides a snapshot of the population of four cancer centres in England and of different oncology dietetic services in DTUs. The main objective of this study was to evaluate whether anthropometric measures of patients attending the DTU for SACT were documented regularly and accurately. Of note, from 15 to 42% of the patients’ weights were not monitored which is a clear area for improvement. Since weights are used to calculate drug dosages and all appointments are in person, it is likely that weights had been measured on the DTU visit days but not documented in the system. This oversight may result from staff shortages or unclear responsibilities regarding who should document anthropometric measurements. Additionally, lack of education in nutrition or cultural factors may lead healthcare professionals to view anthropometric data, like other nutritional information, as unimportant in cancer care compared to medication and disease treatment [3]. However, reliable and frequent documentation of weights could be very valuable for monitoring treatment tolerance and side effects, and would allow prompt referrals for nutrition support [3].

Accuracy of height and BMI documentation also requires improvement, as they are often one of the components of screening tools, such as MUST, which was the tool in use in these cancer centres participating in this study. All centres participating in this audit use electronic notes; centre A and B have both a Trust general system and a DTU specific system for recording DTU data and attendance, whilst centres C and D have a single system. This does not seem to have influenced the results, although in centre A weights are recorded on two different systems, which makes it difficult to visualise weight trends over time. Centres B, C, and D have an online system that automatically calculates BMI when the weight is updated. It can be noted that automation of BMI calculation makes a substantial difference across these centres, as also evidenced in other studies [15–17]. In this audit, automation led to a higher volume and accuracy of documentation by removing human error and workload. Inaccuracies have also occurred in height documentation; for about 15% of patients, different values of height were documented on different dates which could also result in inaccurate BMI calculation. Centre B has an IT system exclusively dedicated to oncology patients, which could explain the lack of inconsistencies which instead occur in centres using IT systems receiving input from several different specialties, centres, and hospitals. A shift is occurring towards digital healthcare and automation in calculating BMI, weight trends, screening scores, and digital flagging of patients at risk should be incorporated in online healthcare system to increase patient safety and monitoring and to support staff [18].

In regard to nutritional screening, although current guidelines recommend its use [4], it was not mandatory in these cancer centres, which likely explains the 1.5% completion rates in this sample. In this cohort alone, 50 patients with a MUST score ≥ 2 had not been referred to a dietitian, which is 13.8% of the population included in this audit. In addition to these 50 patients, there were other 38 patients (9.5%); the authors were unable to screen, which could have potentially increase the number of missed referrals. If we project these numbers on a wider population of three million people with cancer living in the UK currently, better cover may be needed in terms of nutritional services and number of dietitians in cancer centres. The authors reviewed demographic information and calculated MUST scores (Appendix Table 5) for this sample to provide further insights into possible use of screening tools in this population. Demographic information (reported in Table 1 and, more in detail, in Appendix Table 4) reveals how the centres in this study have very similar populations, the median BMI falls within the overweight category across all centres, which reflects a trend observed in the general population BMI [11]. One third of MUST score is determined by BMI (lower BMIs score higher), which may make it a sub-optimal tool for a population which is prevalently overweight. Cancer patients also often suffer from oedema or ascites [20], which would invalidate this type of scoring. This could explain why the calculated malnutrition risk rates of 21.2% in this audit (Appendix Table 5) looks lower than others in literature which use tools more specific for chronic diseases and include some subjective measures as well [12]. A malnutrition risk in 21.2% of this sample could have likely been significantly higher if a more specific tool had been used, such as Patient-Generated Subjective Global Assessment (PG-SGA) or NRS-2002 [4]. Although validated in several populations, MUST is often not considered the most effective tool for screening oncology patients due to the lack of specificity for cancer unique nutritional challenges, such as cachexia and treatment’s side effects. Of note is also the high percentage of human error in MUST calculation, highlighted not only in these results but also by other literature [13, 14].

In regard to dietetic referral rates, results show that centre B has a high percentage of appropriate referrals and a low percentage of missed referrals compared to the other centres, despite all of them not using screening tools regularly. Centre B provided a one-off training session in MUST completion for the staff working in DTU and encouraged staff to use MUST screening. Encouraging and training staff to use MUST does not seem to imply that MUST will actually be performed; however, centre B had the highest documentation rate of weight reported and the least missed referrals of all four centres, therefore, showing a better compliance to monitoring and an increased attention to weight trends. To better interpret these results and further reflect on them, the authors compared referral practices and dietetic team composition across the four centres, as illustrated in Appendix Table 6. From this table, it is apparent that how dietetic staff are distributed and organised to cover different wards and treatment areas varied across centres, as well as the number of dietitians, their specialty, and banding. The proportion of hours dedicated to inpatients and outpatients also is also different across centres, as is the collaboration with other clinical teams, whilst most dietetic referrals across all centres come from specialist nurses, oncologists, and acute dietitians rather than DTU staff. Current practices of oncology dietitians were further discussed with the different teams. From this additional information, it is possible to note that centre B referral criteria are well-defined compared to the other centres and that they occasionally see patients face to face on DTU, which the dietitians in the other centres rarely do. Oncology dietitians in centre B also frequently attend joint clinics and multidisciplinary team (MDT) meetings, which could increase the awareness of other members of staff on nutrition and on the dietetic services available. It is also necessary to note that this audit was conducted during a period of high staff turnover and staff shortages across the NHS, which may partly explain the findings (https://digital.nhs.uk/data-and-information/publications/statistical/nhs-vacancies-survey/april-2015---june-2023-experimental-statistics#chapter-index). In the population analysed in the audit, centres A and B had more than double the number of patients under dietetic care compared to centres C and D. Since most referrals came from non-DTU clinicians, this could be due to a higher multidisciplinary collaboration, or more experienced staff members in some centres, participation in joint clinics or better dietetic staffing levels. The presence of a dietitian in DTU areas may result in increased staff awareness and attention to nutritional care [19]. Despite the better referral rates of centre B, screening would still be recommended to aim at reaching 100% of patients being at nutritional risk.

### Strength and limitations

A strength of this study is its novelty: to our knowledge, no recent study has compared documentation of anthropometrics in populations attending oncology dietetic services in England. Another strength was the collaboration of experienced oncology dietitians from different centres; NHS Trusts tend to have different systems and do not routinely consult each other in terms of comparing services. The population of this study may be a small sample in size in relation to the UK cancer population. However, it likely compares to the situation of several other centres; therefore, this work is relevant for practitioners in England, NHS workers, and patients and is a starting point for further investigation into malnutrition screening and dietetic referrals.

This audit has several limitations, the main one being the difference in team compositions and patient documentation systems, which makes it difficult to compare the service provided across centres. However, the authors have tried to mitigate this by using comparable measures and extensively discussing how to measure the different data. Standardisation was the reason for using MUST ≥ 2 for assessing the appropriateness of the referrals, though the authors acknowledge that appropriate referrals could have included several more or different criteria. If more subjective criteria, such as symptoms affecting oral intake, had been included, the number of missed referrals would likely have been significantly higher. Furthermore, the nature of the audit did not allow for a full nutritional assessment of patients. The audit assessed the use of the MUST tool for screening since it was the tool in use at these centres, but other screening tools might be just as suitable, or even more effective, for a cancer outpatient population. Despite identifying the number of patients receiving dietetic care, this audit does not provide any information on whether these referrals were timely. The ones that should have been referred but were not at the time of this audit may have been missed by the system or may have been referred ‘late’, meaning a period of time after they already started showing signs of malnutrition.

## Conclusions

In the current context, in which holistic care is increasingly prioritised in cancer support, NHS staff cannot afford to overlook the documentation of patients’ weight and height to monitor their nutritional status and intervene as quickly as possible where needed. Furthermore, leveraging existing technology and automation becomes even more critical, especially in light of the recent NHS short-staffing issues. By fostering collaboration and ongoing discussions among teams working in different geographical areas, it becomes possible to determine common standards for patient care at a national level. Such collaboration could also help highlight successful initiatives in different areas and the development of common strategies. From the results of this study, dietetic referral criteria and interdisciplinary collaboration seem critical in being able to provide nutritional care to the patients in need. Therefore, further research should prioritise investigating and defining the criteria and timings of providing optimal and equal nutritional care to all patients, ensuring cancer centres take a proactive approach in offering support.

## Data Availability

No datasets were generated or analysed during the current study.

## Appendix

**Table 4:** Sample demographics for each individual centre and overall

	Centre A	Centre B	Centre C	Centre D	Overall
Mean weight in kgs	74.4	77.1	76.7	76.4	76.5
Median weight in kgs	72.2	75.4	70.8	73.4	73
Range of weights in kgs	43.9–119.8	45–125.6	46–127	32–121	32–127
Mean age in years	61	63	64	61	62
Median age in years	63	66	66	62	65
Ranges of age in years	20–85	33–85	33–85	27–88	20–88
Ratio M:F (No.)	42:58	31:69	39:61	41:59	153:247 (38.25% and 61.75%)
Self-described ethnicity (No.)	White: 64 Other: 32 Black: 0 Asian: 2 Prefer not to say: 2	Data not available	White: 53 Other: 16 Black: 1 Asian: 13 Prefer not to say: 17	White: 61 Other: 0 Black: 11 Asian: 5 Prefer not to say: 23	White: 178 (59.33%) Other: 48 (16%) Black: 12 (3.6%) Asian: 20 (6.66%) Prefer not to say: 42 (14%)
Cancer diagnosis (No.)	H&N: 7 UGI: 5 HPB: 4 Lung: 6 Breast: 21 Gynae: 8 Pelvic: 4 LGI: 7 Haem: 24 Melanoma: 8 Others: 6	H&N: 7 UGI: 1 HPB: 14 Lung: 11 Breast: 25 Gynae: 9 Pelvic: 4 LGI: 24 Haem: 0 Melanoma: 5 Others: 0	H&N: 0 UGI: 1 HPB: 6 Lung: 5 Breast: 20 Gynae: 12 Pelvic: 9 LGI: 24 Haem: 21 Melanoma: 0 Others: 2	H&N: 3 UGI: 8 HPB: 16 Lung: 11 Breast: 12 Gynae: 14 Pelvic: 5 LGI: 18 Haem: 0 Melanoma: 4 Others: 9	H&N: 17 (4.25%) UGI: 15 (3.75%) HPB: 40 (10%) Lung: 33 (8.25%) Breast: 78 (19.5%) Gynae: 43 (10.75%) Pelvic: 22 (5.5%) LGI: 73 (18.25%) Haem: 43 (10.75%) Melanoma: 17 (4.25%) Others:19 (4.75%)
Mean BMI in kgs/m^2^	27	27.6	28.7	26.9	27.7
Median BMI in kgs/m^2^	29	26.9	27.4	25.7	26.8
Range of BMIs in kgs/m^2^	17.2–46.7	16.5–41.2	18.8–42.7	13–46	13–46.7

**Table 5:** MUST scores calculated by the authors in the audit sample

	Centre A	Centre B	Centre C	Centre D	Total
Patients scoring MUST ≥ 2—No. (%)	24/87 (27.5)	13/87 (15)	16/96 (16.6)	24/92 (26)	77/362 (21.2)
MUST 0—No. (%)	50/87 (57.4)	73/87 (83.9)	57/96 (59.3)	57/99 (57.5)	237/362 (65.4)
MUST 1—No. (%)	13/87 (14.9)	1/87 (1.1)	23/96 (23.9)	11/99 (11.1)	48/362 (13.2)
MUST 2—No. (%)	14/87 (16.0)	7/87 (8.0)	13/96 (13.5)	17/99 (17.1)	51/362 (14.0)
MUST 3—No. (%)	6/87 (6.8)	4/87 (4.5)	3/96 (3.1)	3/99 (3)	16/362 (4.4)
MUST 4—No. (%)	3/87 (3.4)	2/87 (2.2)	0/96 (0)	2/99 (2.0)	7/362 (1.9)
MUST 5—No. (%)	1/87 (1.1)	0/87 (0)	0/96 (0)	0/99 (0)	1/362 (0.2)
MUST 6—No. (%)	0/87 (0)	0/87 (0)	0/96 (0)	2/99 (2.0)	2/362 (0.5)
Patients for whom it was not possible to calculate MUST due to lack of data—No. (%)	13 (13)	13 (13)	4 (4)	8 (8)	38/400 (9.5)

**Table 6:** Estimates of dietetic service composition and practices among the cancer centres

	Centre A	Centre B	Centre C	Centre D
N. of patients attending DTU in a typical working day	≈200	≈110	≈306	Not known
N. of patients attending DTU to receive treatment	≈60	≈60	≈70	≈60
Dietitians’ hrs dedicated to outpatients per week	≈123	≈75	≈45	≈52
Dietetic oncology team members	DA: 0.5 WTE Band 5: 1.5 WTE Band 6: 5 WTE Band 7: 4.5 WTE Band 8: 0	DA: 0.8 WTE Band 5: 0 Band 6: 1 WTE Band 7: 1.9 WTE Band 8: 0	DA: 0 Band 5: 1 WTE Band 6: 3.3 WTE Band 7: 1.5 WTE Band 8: 0.3 WTE	DA: 0.4 WTE Band 5: 1 WTE Band 6: 2.4 WTE Band 7: 1.8 WTE Band 8: 0
Vacant roles at the time of data collection	WTE band 5 WTE band 7 0.5 WTE band 6	Nil	Band 7 0.5 WTE Band 6 1.3 WTE Band 5 1 WTE	Band 6 0.4 WTE
Screening used in DTUs	None	MUST encouraged	None	None
Dietetic referral criteria	Not clearly defined	MUST score 2 + , dysphagia, any symptoms affecting intake	Not clearly defined	Not clearly defined
Dietetic referrals system	Message on online system (Citrix – EPR)	Paper form filled in/scanned in and emailed	Electronic via email	Through online system PICS. Occasionally by email
N. of outpatients’ referrals per week	≈12–16 patients	≈10–15 patients	≈9.3 patients	≈0.5 patients
Who refers outpatients	Specialist nurses and DTU nurses, oncologists, dietitians	Outpatient units, clinical nurse specialists, oncologists, acute dietitians	Specialist nurses, GPs, oncologists, AHPS (SLT/OT), community dietitians, DTUs	Clinical nurse specialists, oncologists, other AHP’s, community dietitians and GP’s
